# Role of Stem Cell Factor in the Reactivation of Human Fetal Hemoglobin

**DOI:** 10.4084/MJHID.2009.009

**Published:** 2009-11-13

**Authors:** Marco Gabbianelli, Ugo Testa

**Affiliations:** Department of Hematology, Oncology and Molecular Medicine, Istituto Superiore di Sanità, ROME, ITALY

## Abstract

In humans the switch from fetal to adult hemoglobin (HbF → HbA) takes place in the perinatal and postnatal period, determining the progressive replacement of HbF with HbA synthesis (i.e., the relative HbF content in red blood cells decreases from 80–90% to <1%). In spite of more than twenty years of intensive investigations on this classic model, the molecular mechanisms regulating the Hb switching, as well as HbF synthesis in adults, has been only in part elucidated. In adult life, the residual HbF, restricted to F cell compartment, may be reactivated up to 10–20% of total Hb synthesis in various conditions associated with “stress erythropoiesis”: this reactivation represented until now an interesting model of partial Hb switch reverse with important therapeutic implications in patients with hemoglobinopathies, and particularly in β-thalassemia. *In vitro* and *in vivo* models have led to the identification of several chemical compounds able to reactivate HbF synthesis in adult erythroid cells. Although the impact of these HbF inducers, including hypomethylating agents, histone deacetylase inhibitors and hydroxyurea, was clear on the natural history of sickle cell anemia, the benefit on the clinical course of β-thalassemia was only limited: particularly, the toxicity and the modest increase in γ-globin reactivation indicated the need for improved agents able to induce higher levels of HbF. In the present review we describe the biologic properties of Stem Cell Factor (SCF), a cytokine sustaining the survival and proliferation of erythroid cells, that at pharmacological doses acts as a potent stimulator of HbF synthesis in adult erythroid cells.

## Introduction:

Worldwide, thalassemia is one of the most common genetic diseases. It affects peoples who originate from the Mediterranean areas through the Middle East to the Indian subcontinent and Southeast Asia^1^. The thalassemia syndromes are broadly classified into two groups: α-thalassemia, in which the α-globin chain synthesis is defective; and β-thalassemia, which results from defective β-globin chain synthesis. α-thalassemia is most commonly caused by gene deletions that affect one or both of the duplicated α-globin loci. The severe form of α-thalassemia, common in Southeast Asia, is caused by deletion of the duplicated α-globin genes, and the majority of homozygous fetuses die at the third trimester of pregnancy or at birth. In β-thalassemia the common molecular defects are the results of some mutations (point mutations or small deletions) that affect the transcription, splicing, or translation of the mRNA. Newborns with homozygous β-thalassemia are healthy, but as the hemoglobin switches from fetal to adult hemoglobin synthesis just after birth, the lack of β-globin chain synthesis results in the development of progressive anemia. In these patients the degree of anemia may vary depending on the degree of β-globin chain deficiency, but the majority of them have severe anemia requiring life-long blood transfusions and, consequently, iron chelation. Hence, β-thalassemia represents a severe health problem and many therapeutic strategies have been attempted.

Cure of β-thalassemia can be achieved by bone marrow or cord blood transplantations if histocompatible donors are available^2^. However, since these patients’ families are usually small, perfectly histocompatible donors are not commonly found. In addition, graft versus host disease of variable severity usually follows and complicates the clinical course of these transplantations. The other approach of curative treatment of β-thalassemia consists in the gene therapy, but this approach is only at the beginning and till now very few patients have been treated^3^; furthermore, this treatment is not on the point of receiving a marked clinical development in the next years.

An alternative treatment of β-thalassemia consists in the pharmacological stimulation of fetal hemoglobin (HbF) synthesis. In humans, hemoglobin switch from HbF to adult hemoglobin (HbA) occurs in the period around birth as a result of γ- to β-globin gene switching. This switching requires developmental stage-specific changes in the expression/function of transcription factors and chromatin remodeling activities that induce repression of γ-globin gene expression and/or induction of β-globin gene expression^4^,^5^. HbF is synthesized at very low levels in adults, representing less than 1% of total hemoglobin^6^. Moreover, the level of HbF is inherited as a quantitative trait and is of enormous clinical relevance, given its role in ameliorating the severity of the main hemoglobin diseases, including β-thalassemia and drepanocytosis. Recent genome-wide association studies have identified three major loci containing a set of five common single-nucleotide polymorphisms (SNPs) that account for ≈20% of the variation in HbF levels^7^–^9^. Importantly, several of these variants predict the clinical severity of sickle cell disease^7^ and of β-thalassemia^8^. The SNP with the largest effects on HbF synthesis is located in the second intron of a gene present on chromosome 2, encoding BCL11A. A recent study has provided clear evidence that BCL11A is an important regulator of HbF expression. BCL11A acts as a repressor of HbF synthesis and its expression, is low in fetal erythroid cells, is abundant in adult erythroid cells; downregulation of BCL11A in adults erythroid cells leads to a remarkable HbF expression^10^. Another SNP that plays an important role in the control of HbF synthesis is located at the level of HBS1L-MYB intergenic region on chromosome 6q23: this locus contains regulatory sequences that play an important role in the control of HbF synthesis and hematopoiesis by controlling MYB expression^11^.

The γ-globin gene transcription is controlled by complex molecular mechanisms involving both cis-acting elements, represented by specific nucleotide sequences, such as Locus Control Region (LCR) and trans-acting elements, such as transcription factors and chromatin remodeling activities^4^. The human β-globin LCR was functionally defined as a DNA regulatory region conferring high levels of erythroid-speciifc expression to a cis-linked gene in a copy-dependent manner, which is independent of the integration site. GATA-1, EKLF and NF-E2 are the best known tissue-specific transcription factors involved in the transcriptional control of β-like globin genes. GATA-1 and EKLF transcription factors are essential for an active chromatine hub (ACH) formation.

Important informations on cis-elements regulating γ-globin gene expression derive from the analysis of the mutations occurring at the β-globin locus in patients with increased HbF (Hereditary Persistance of Fetal Hemoglobin) (reviewed in 12). Two types of HPFH have been identified: non-deletion and deletion-type HPFH. Non-deletion HPFHs are due to mutations occurring in either the ^G^γ or ^A^γ-globin gene promoters, thus resulting in continued HbF synthesis in adult life. These non-deletion HPFH mutations are clustered in three regions of the γ-globin promoters, at the level of the positions -200, -175 and -115 relative to the transcription site^13^. The increased HbF synthesis observed in adults carrying non-deletion HPFH mutations are due to either a decreased binding of transcription repressors inhibiting γ-globin gene expression or to the generation of binding sites that potentiate the binding of a transcriptional complex increasing γ-globin gene transcription. On the other hand, the analysis of the DNA deletions as well as of the phenotype of deletion-type HPFH has provided important information’s on the existence of DNA sequences of the β-globin gene cluster affecting γ-globin gene expression. In the African type of deletion HPFH, large deletions have been observed involving intergenic γ-δ sequences and the entire β and δ genes. The phenotype of these HPFH forms is seemingly related to the juxtaposition of enhancer elements, normally located downstream of the globin locus, and the HPFH breakpoint exerting a positive stimulatory effect on γ-globin transcription^14^. The analysis of the gene deletion occurring in δβ thalassemia and, particularly in δβ° Corfu deletion strongly supports the notion that intergenic γ-δ sequences play a key role in the adult γ-globin reactivation.

In addition to genetic elements present in the β-globin locus, also genetic elements present outside this locus control the level of HbF synthesis during the adult life^7^–^10^. These genetic elements contribute to inter-individual variation in HbF expression representing a heritable disease modifier^15^. Three major loci – Xmn1-HBG2 single nucleotide polymorphism, HBS1L-MYB intergenic region on chromosome 6q, and BC11A- contribute to HbF variation in healthy European Caucasians and β-thalassemia and sickle cell traits^15^. These HbF quantitative trait loci and others not yet discovered, besides accounting for the common HbF variation in healthy adults, play also an important role in the variation of HbF production in response to erythropoietic stress, and possibly, in the capacity to respond to pharmacological stimulators of HbF synthesis^16^. It is of interest to note that two of these quantitative trait loci include oncogenes, emphasizing the importance of cell proliferation and differentiation as an important contribution to the HbF phenotype^17^.

Finally, an important role in the control of globin gene expression is played also by chromatin epigenetic changes. The main chromatin epigenetic changes occur at the level of core histones and consist in covalent modifications such as acetylation, methylation and phosphorylation. Among these modifications a relevant role is played by histone acetylation in the control of γ-globin gene expression: active γ-globin genes are highly acetylated, while reactive γ.globin genes are scarcely or only midly acetylated^18^. Histone methylation plays also an important role in γ-globin gene expression: a low histone methylation at the level of the γ-globin gene promoter is associated with elevated HbF synthesis, while a high level of histone methylation is linked to repression of HbF synthesis^19^.

## Chemical inducers of HbF synthesis:

The analysis of the molecular mechanisms underlying hemoglobin switching and, particularly γ-globin gene expression, have greatly contributed to the identification, study and possible clinical development of chemical compounds able to reactivate HbF synthesis in adult erythroid cells. A fundamental input to the search of efficient HbF chemical inducers came from the observation, made several decades ago, that HbF may have a protective effect in sickle cell anemia and β-thalassemia^20^.

During the last 30 years a considerable number of pharmacological agents able to reactivate HbF synthesis have been identified. Some of these agents have been introduced for the treatment of sickle cell anemia and β-thalassemia. According to their mechanism of action these compounds have been classified in various groups: hypomethylating agents, histone deacetylase inhibitors and hydroxyurea. For the analysis of the chemical structure and the biologic properties of these HbF inducers the readers may refer to some recent reviews^21^,^20^,^22^.

The past experimental and clinical observations in the treatment of hemoglobinopathies supported the basic conception that these chemical agents represent a rational approach for sickle cell anemia and β-thalassemia therapy. However, it must be pointed out that, while the effect of these pharmacological treatments on sickle cell anemia was clear, their benefit on the clinical course of β-thalassemia was only limited. The discrepancy between these two hemoglobinopathies in the response to HbF inducers may be mainly related to the higher level of HbF required in β-thalassemia to achieve clinical results comparable to those observed in sickle cell anemia. Furthermore, it was recently suggested that the limited clinical response to γ-globin inducers, observed in the majority of β-thalassemic patients, may be also a reflection of unfavorable effects of these agents on the other globin genes (i.e., increased α-globin synthesis)^23^. This observation suggests that other agents able to induce higher HbF levels are required for an efficient β-thalassemia treatment.

Recently, the gene therapy approach was also used to increase the level of HbF synthesis in β-thalassemic erythroid cells. Particularly, it was shown that amelioration of a murine model of β-thalassemia can be achieved through *in vivo* drug selection of hematopoietic stem cells transduced with a dual γ-globin/methylguanine methyltransferase lentiviral vector^24^.

In the present review we describe the biologic properties of Stem Cell Factor (SCF also known as Kit Ligand), a cytokine that, besides sustaining survival, stimulating hematopoietic progenitor cell proliferation and enhancing proliferative response to erythropoietin, at pharmacological doses acts as a potent stimulator of HbF synthesis in adult erythroid cells.

## Role of SCF in the control of erythropoiesis:

Erythropoiesis is subject to a complex regulation via a network of cytokine receptor systems that act at the level of various stages during erythroid differentiation and maturation. Erythopoietin (Epo) is an essential key regulator of erythropoiesis, mainly acting as a cytokine promoting the survival and proliferation of late erythroid progenitors (CFU-E). The growth of early erythroid progenitors (BFU-E) is promoted by various interleukins, including IL-3, IL-11, IL-4 and IL-12. In addition, erythropoiesis is regulated by a series of receptor tyrosine kinases (RTKs). Among them, a relevant role is played by RON (c-met-related tyrosine kinase) and its ligand that exert a stimulatory effect on BFU-E proliferation^25^, Insulin-Like-Growth Factor and its receptors stimulating late stages of erythropoiesis^26^ and EPHB4 that exerts a stimulatory effect on erythropoiesis at various differentiation stages^27^.

Particularly, an important role in the control of erythropoiesis is played by SCF and its receptor (SCF-R or c-kit). The initial observations leading to discover a role of this cytokine in erythropoiesis derived from the analysis of mice exhibiting mutations in the SCF gene, Steel (Sl), or its receptor c-kit: these mice develop severe anemia characterized by depressed erythropoiesis. Particularly, mice that lack the expression of c-kit (the c-kit white spotting mutant, c-kit^w^, encodes a shortned protein lacking the transmembrane portion, which therefore, fails to be expressed on the cell surface) exhibit a severe reduction of CFU-E number in the fetal liver and die of anemia around day 16 of gestation^28^. Importantly, the transgenic expression of Epo can overcome the lethality caused by the c-kit^w/w^ mutation: in the mutant mice rescued by Epo, CFU-Es are rescued to normal frequencies^29^. This important observation suggests that EpoR signals can partially bypass the strict requirement for c-kit signaling in erythropoiesis in the absence of c-kit *in vivo*^29^.

SCF belongs to the family of 4-helix-bundle cytokines and growth factors, forming a non-covalently associated dimmer, similar in structure to M-CSF and Flt3L. SCF is synthesized as a transmembrane-anchored precursor. SCF mRNA is alternatively spliced, forming two different transmembrane precursors, distinguishable on the basis of their sensitivity to cell-surface proteases: (a) the larger SCF-M1 contains a proteolytic site and generates soluble SCF; (b) the smaller SCF-M2 lacks this proteolytic site, giving rise to a membrane-bound form. This SCF membrane-bound form is mainly involved in the control of hematopoiesis.

The receptor for SCF, c-kit, is a type III receptor tyrosine kinase that belongs to the same subfamily as PDGFR, Flt3R, M-CSFR. C-kit is expressed in the gastrointestinal system, in melanocytes and in germ cells. Stimulation of the c-kit receptor with its ligand SCF, leads to receptor dimerization and activation of its intrinsic tyrosine kinase activity.

C-kit is expressed on the majority of CD34+ hemopoietic progenitors and its expression is maintained at high levels during the stages of differentiation from BFU-E to CFU-E; at later stages of differentiation during the maturation of CFU-E, c-kit expression progressively declines and disappears in polychromatophilic and orthochromatic erythroblasts^30^. These findings imply that SCF may exert effects on erythroid cells during early and late stages of erythroid differentiation and may act also on immature erythroid precursors^30^. Repression of c-kit expression and signaling at late stages of erythroid differentiation is mediated by the transcription factor GATA-1 through direct interaction with the c-kit gene promoter^31^. Particularly, it was shown that during early stages of erythroid differentiation the c-Kit gene promoter is occupied by the GATA-2 transcription factors that exerts a stimulatory role on the transcription of this gene; upon cell differentiation, GATA-1 displaces GATA-2 and triggers a repressive effect on c-kit gene transcription^32^. Other studies have shown that the transcription factor PLZF is required, together with GATA-2, for allowing elevated level of c-Kit expression during early stages of erythroid differentiation^33^ and the microRNA 221 and 222 are involved in c-kit downmodulation occurring at late stage of erythroid differentiation^34^.

SCF/c-kit interactions lead to c-kit autophosphorylation at the level of up to 21 c-kit phospho-tyrosine (PY) docking residues for SH2 and PTB domain proteins. Using suitable cell line models, studies on c-kit PY mutants have defined the specificities of these PY residues: Y719 binds p85-PI3K; Y728 binds phospholipase C-γ; Y702 and Y934 bind Grb2 and Grb7 adaptor binding proteins; juxtamembrane Y567 and Y569 sites bind SRC family kinases and, through this mechanism, activate key survival and proliferation pathways. To define the biologic function of the various PY residues of c-kit, mice carrying tyrosine-to-phenylalanine substitution mutations have been generated. These studies have shown that Y569 and Y719 residues are not essential for erythropoiesis. However, a recent study provided evidence that in kit^Y567F/Y567F^ mice, steady state erythropoiesis was unperturbed, while recovery from chemically-induced anemia was markedly impaired, thus indicating a key role of c-kit in the control of stress erythropoiesis^35^.

These observations clearly showed that c-kit signalling is important for normal erythropoiesis and, particularly for stress erythropoiesis. On the other hand, numerous *in vitro* and *in vivo* experiments have shown that SCF and Epo co-administration results in a stimulation of erythropoiesis and BFU-E and CFU-E colony formation: particularly, *in vitro* administration of SCF together with Epo resulted in a marked enhancement of erythroid proliferation, associated with a delay in erythroid maturation.

Other studies have suggested that SCF could potentiate the stimulation of some transduction pathways activated by Epo. The studies aimed at elucidating the synergistic interaction between SCF and Epo have proposed two main underlying mechanisms. The first mechanism suggests that the synergism is mainly related to the sequential role of SCF and Epo during erythroid differentiation. The second mechanism suggests that SCF and Epo have simultaneous, joint effects on the same individual cells at a specific stage of erythroid development (proerythroblast stage). In this context, recent studies support a model of erythroid differentiation in which the effects of SCF and Epo on individual erythroid progenitors/precursors are mainly sequential and co-signaling (i.e., signaling by both cytokines on the same cells, resulting or not in crosstalk between pathways) has only a limited contribution to the final erythroid output^36^.

Kit and EpoR mediated co-signaling is essential for normal erythroid cell expansion; however, the intracellular signals that contribute to cooperative signaling have been only in part elucidated. Studies with mutants of c-kit and EpoR suggest that kit and EpoR mediated co-signaling requires intracellular tyrosine residues: particularly, tyrosine residues that bind Src kinases in the kit receptor (i.e., Y^567^ and Y^569^) appear to be sufficient for kit signaling as well as co-signaling with EpoR^37^.

Activation of PI3K is essential for proliferation of erythroid cells. Activated PI3K generates PIP3, which serves as an anchor for multiple PH-domain-containing proteins, such as protein kinase B (PKB). Although both SCF and Epo induce activation of PI3K in erythroblasts, the efficiency with which respective downstream signaling pathways are activated shows large differences: particularly, the activation of PKB is more responsive to SCF than to Epo^38^,^39^. PI3K stimulates the activation of mTOR and subsequent release of the cap-binding translation initiation factor 4E (eIF4E) from the 4E-binding protein 4EBP, which controls the recruitment of structured mRNA to polysomes. Enhanced expression of the eIF4E renders erythroblast proliferation independent of PI3K. The activation of eIF4E by SCF determines the selective association with polysomes of some mRNAs, whose translation is then stimulated. One of these mRNAs is represented by Immunoglobulin Binding Protein 1 (IGBP1): this gene is involved in regulating erythroid progenitors renewal versus differentiation. In fact, the overexpression of this gene in erythroid cells mimics the effect of SCF, impairing differentiation and promoting erythroid proliferation^40^.

SCF and Epo synergistically activate MAP kinase (MAP ERK1/2), acting through different molecular mechanisms^41^. Studies carried out on marrow CFU-E provided evidence, however, that Epo alone resulted in a weak ERK activation, while SCF alone elicited a clear stimulation of ERK activity; the co-administration of both cytokines resulted in a marked induction of ERK activity^42^. Interestingly, the stimulatory activity on ERK activity exerted by the membrane-bound form of SCF was more pronounced than that elicited by the soluble form of SCF^43^; in line with this observation, the membrane-associated SCF stimulated erythropoiesis more efficiently than the soluble SCF form. In line with these observations recent studies performed on *in vitro* generated CFU-Es provided clear evidence that SCF, but not Epo, is able to induce the phosphorylation of MEK^43^. This MEK activation does not seem to be involved in the stimulatory effect of SCF on erythroid cell proliferation, but is involved in mediating specific pathway of gene activation induced by SCF^44^.

Furthermore, SCF stimulates EpoR expression and Stat5 expression, although it was unable to phosphorylate and then to activate this transcription factor^45^. Recent studies confirmed that SCF was unable to activate Stat-5 phosphorylation and products of Stat-5 target genes induced by Epo act to enhance c-kit signalling^46^. Stat-5 seems to be essential for stress erythropoiesis and for mediating the response to anemia: EpoR and KIT-induced Stat5 signals induce factors (such as Bcl-x and oncostatin-M) required for the survival of erythroid progenitors/precursors^47^.

Recent studies suggest that, in addition to Epo and SCF, also glucocorticoids have a remarkable effect on erythropoiesis. These hormones act through nuclear receptors and recent investigations indicate that mice deficient in glucocorticoid receptor expression exhibit a complete loss of stress erythropoiesis, i.e., the capacity to respond with an increased erythropoietic production to stress conditions, such as erythrolysis or hypoxia^48^. *In vitro* studies have shown that glucocorticoids are able to exert a marked stimulatory effect on erythropoiesis: their effect, however, is evident only when erythroid cells are grown in the presence of both Epo and SCF^49^,^50^. The molecular mechanisms responsible for the stimulatory role of glucocorticoids on erythropoiesis are not precisely determined, but they seem to involve an up modulation of c-kit and c-myb expression^51^. Interestingly, p53 antagonizes the stimulatory activity of glucocorticoids on erythropoiesis^51^.

The mechanism of the cooperative signaling between Epo, KL and glucocorticoids was explored by micro array profiling. This type of analysis provided evidence that the majority of the genes are modulated by glucocorticoids only in the presence of Epo and KL, suggesting that these hormones function in the context of large transactivation complexes. The basic effect of glucocorticoids seems to be mainly related to the induction of genes involved in cell renewal and to the attenuation of genes exerting a growth-inhibitory effect^52^. In addition to glucocorticoids also androgens exert a stimulatory effect on erythroid proliferation; however, since normal erythroblasts lack androgen receptors, the stimulatory effect on these receptors on erythropoiesis seems to be related to the usurpation of other nuclear hormone receptors by androgens^53^.

The rapid response to anemia does not involve only SCF, but also Bone Morphogenetic Protein 4 (BMP4). BMP4 is rapidly, and induced erythropoiesis cells in the red splenic pulp of anemic animals in response to acute anemia and was found to stimulate immature progenitors to give rise to Epo-responsive progenitors. Studies in animal models have shown that erythroid response to acute anemia involves the rapid expansion of a specialized population of erythroid progenitors, termed stress BFU-E, mediated by BMP4^54^. SCF and hypoxia synergize with BMP4 to promote the expansion and differentiation of stress BFU-E during the recovery from acute anemia^55^. More recently, it was demonstrated that the response of erythroid progenitors to BMP4 is regulated by hedgehog signaling^56^.

## Effect of SCF Factor on Hb F synthesis:

Considering the pronounced effects of SCF on erythropoiesis it seemed important to evaluate a possible effect of this cytokine on HbF synthesis. The majority of these studies have been carried out by investigating SCF effect in adult erythroid cell cultures.

In this regard, the evaluation of cytokine effects on erythroblast HbF synthesis requires a stringent methodology, involving the use of purified hemopoietic progenitors (usually human CD34^+^ purified cells) and serum-free conditions^57^–^60^. In fact, fetal calf serum, routinely used for culture of human hemopoietic cells, contains factors able to to stimulate HbF synthesis^57^–^60^. Furthermore, accessory cells present in cultures of unpurified hemopoietic progenitors, may affect or modify the effect of exogenous cytokines on HbF synthesis.

In an initial study Miller et al. observed a clear stimulation of HbF synthesis induced by SCF in normal (from 0.5% to 6%) and sickle cell anemia (from 4 to 6.8%) BFU-Es grown under serum-free conditions^61^. These findings were confirmed and extended by Peschle et al. using purified hemopoietic progenitors grown under serum-free conditions either in semisolid medium to generate BFU-E or in liquid suspension cultures allowing the selective growth of erythroid cells^62^. Using these unilineage culture conditions it was observed that SCF at optimal concentrations (i.e., 100 ng/ml) elicited a marked enhancement of HbF synthesis levels up to 20%^62^. The stimulatory effect of SCF on HbF production could be mediated either by an effect on the BFU-E HbF synthesis program or by the recruitment of BFU-Es with an elevated HbF production potential. Experiments carried out on single BFU-Es provided evidence in favor of a direct, dose-related stimulatory effect of SCF on HbF synthesis reactivation^63^. In this same study it was shown that SCF was able to synergize with sodium butyrate to enhance HbF synthesis: in fact, the combined addition of the two agents together induced HbF synthesis levels up to 40–43% in normal adult erythroid cells^63^.

Wojda et al. comparatively analyzed twelve cytokines acting on erythropoiesis and confirmed that, among them, only SCF was able to significantly enhance HbF synthesis^64^. The capacity of SCF to reactivate HbF synthesis in adult erythroblasts was correlated to the presence of c-kit on erythroid progenitors/precursors: in fact, the delayed addition of SCF to erythroid cultures was active in inducing a marked enhancement of HbF synthesis only if added on erythroid cell progeny c-kit^+64^. Subsequent studies have shown the synergistic effect between SCF and other agents in stimulating HbF synthesis. Two molecules, Transforming Growth Factor-β (TGF-β) and corticosteroids, potentiate the effect of SCF on HbF synthesis. Thus, despite their opposite effects on erythroid cell growth, SCF and TGF-β had synergistic effects with respect to HbF synthesis^65^. In erythroid cultures supplemented with Epo+SCF+TGF-β about a 40% of HbF synthesis was observed, with a pancellular distribution of HbF in erythroid cells^65^. The evaluation of glucocorticoids effects on HbF synthesis, alone or in cooperation with SCF, was prompted from studies showing that corticosteroids, such as dexamethasone, cooperate with Epo and SCF to enhance and sustain the proliferation of erythroid progenitors^66^. Dexamethasone when added together with Epo and SCF enhanced HbF synthesis up to 55%; furthermore, analysis of erythroid cultures of sibling BFU-Es showed that the stimulatory effect of Dexamethasone+SCF was related to the modulation of γ-globin expression rather than to recruitment of BFU-Es with elevated HbF synthetic potential^67^.

The effect of SCF was recently investigated in cultures of β-thalassemic erythroid cells. In erythroid cultures of β-thalassemic patients, addition of SCF (in the presence of Epo), remarkably stimulated cell proliferation (3–4 logs over control cultures), decreased the percentage of apoptotic and dyserythropoietic cells and markedly increased γ-globin synthesis, reaching levels 3-fold higher than in control cultures (from 27% to 81%)^65^. These studies indicate that in β-thalassemia SCF induces an expansion of effective erythropoiesis and the reactivation of HbF synthesis up to fetal levels and may hence considered as a therapeutic agent for this disease^68^.

The mechanisms through which SCF stimulates HbF synthesis in adult erythroid cells is largely unknown. It is evident that the mechanism of HbF induction by SCF must be related to one or several signaling pathways induced by the interaction of this cytokine with its receptor c-kit. In this context, Bhanu et al^69^ evaluated the effect of inhibitors of various signaling pathways, including JAK2, PKC, PI3K, p38MAPK, guanylate cyclase and MEK inhibitors. Only MEK inhibitors elicited a pronounced inhibitory effect on HbF synthesis, thus indicating that phosphorylation of ERK-1/ERK-2 MAPK plays an essential role in the mechanisms through which SCF activates γ-globin gene expression in adult erythroid cells. At variance with SCF, the large majority of the other HbF chemical inducers activate p38 MAPK cell stress signaling pathway and this pathway is required for their stimulatory effect on HbF synthesis^70^.

Among the large number of proteins binding to the γ-globin promoter region, it was recently demonstrated that stage-specifically expressed nuclear receptor chicken ovalbumin upstream promoter- transcription factor II (COUP-TFII) and the direct repeat erythroid definitive binding protein TR2/TR4, are implicated in SCF-mediated HbF reactivation^67^. Particularly, it was shown that COUP-TFII expression is suppressed by SCF through phosphorylation of serine/threonine phosphatase and this event is required for SCF-induced HbF reactivation^71^. This finding may be linked to the previous studies on the role of ERK1/2 on SCF-mediated HbF induction. In fact, SCF, through stimulation of ERK1/2 MAPK signaling pathway, regulates the downstream repressor COUP-TFII by inhibiting serine/threonine phosphatase 2A activity and the decreased COUP-TFII expression resulted in γ-globin reactivation^71^.

Other studies have suggested a possible role of some transcription factors whose expression is induced by SCF, such as Id2, Tal-1 and FKLF in mediating the transcriptional activation of the human γ-globin gene 63,67. A recent study provided evidence that SCF treatment of adult erythroid progenitors was associated with consistent changes of globin gene histones and with a concomitant transcriptional activation pathway involving reproducible changes in expression of some nuclear transcription factors that are recognized regulators of erythropoiesis or globin genes (MAFF, Id2, HHEX, SOX6 and EGR1)^72^. Interestingly, in this thudy an inhibitory effect of SCF on the expression of BCL11A mRNA expression was reported^72^.

These SCF *in vitro* observations must be extended to *in vivo* studies in order to evaluate optimal doses, schedules of administration and side effects. In this context, combined doses of SCF and Epo have been administered to baboons, achieving up to 20% HbF levels^73^. Preclinical studies in primates and mice suggested that SCF occasionally causes significant allergic side effects, related to induction of mastocytosis^74^,^75^. This was confirmed by subsequent clinical trials in HIV and cancer patients^76^,^77^, aplastic anemia^78^, cord blood transplantation^79^ and postablative chemotherapy^80^: specifically, mild allergic reactions (e.g., pruritus, urticaria, cutaneous angioedema) were reported in a minority of patients, while severe ones (e.g., laringospasm) were observed only in exceptional cases. In the most extensive phase 3 trial, SCF was used for stem cell mobilization: patients received recombinant SCF at 20 μg/kg per day for 5 consecutive days and 3 of them reported systemic allergic reactions, resolved after treatment with steroids^81^. The potential use of SCF for treatment of β-thalassemic or sickle cell anemia patients implies a chronic administration protocol, based on sequential intermittent therapy cycles: this protocol may amplify the potential risk of allergic reactions and must be considered cautiously in view of these possible side effects.

It is of interest to note that recent studies have involved the SCF/c-kit system in the mechanism of physiologic hemoglobin switching. First, it was observed that the level of c-kit and SCF expression is particularly elevated during embryonic/fetal life at the level of tissues involved in hematopoiesis (AGM region and fetal liver)^82^. Second, it was reported that cord blood serum contains clearly higher SCF levels than adult serum, a phenomenon seemingly related to the capacity of human umbilical vein endothelial cells to release large amounts of SCF^83^–^85^. Third, c-kit expression on cord blood CD34^+^ cells gradually declines: this decrease is directly related to the decline of HbF content. This decline of c-kit expression is paralleled by a concomitant gradual decrease of SCF levels (Gabbianelli M et al, unpublished observations). Altogether these observations suggest that a decline of kit activity could play a role in the mechanism of perinatal HbF→HbA hemoglobin switching. In line with this hypothesis, addition of SCF in cord blood BFU-E cultures reactivates HbF production in a dose-dependent fashion, almost up to the pre-switch levels (Gabbianelli M et al, unpublished observations). The mechanisms underlying the modulation of c-kit expression during ontogenesis could be, at least in part, related to the c-kit suppressing miR221/miR222 complex, whose levels increased during development (Gabbianelli et al, unpublished observations). Interestingly, a recent study by Bianchi et al^86^ suggested that the hypoxia-associated miR210 might be involved in increased expression of γ-globin genes in differentiating erythroid cells. At the moment it is unknown whether miR210 expression is controlled by SCF and is modulated in erythroid cells during ontogenesis, and particularly during the Hb switching period.

## Figures and Tables

**Figure 1.**
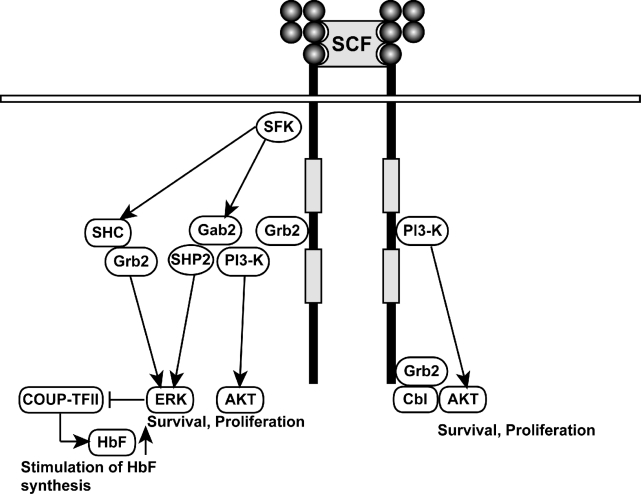
Schematic representations of the SCF/c-kit cell signaling. The main pathways activated by the activation of the c-kit receptor are outlined. The pathways seemingly involce in HbF reactivation are outlined.
